# Supplementary material for: Role of Uric Acid in Vascular Remodeling: Cytoskeleton Changes and Migration in VSMCs
Source: Int J Mol Sci. 2023 Feb 3;24(3):2960. doi: 10.3390/ijms24032960 (PMC9917405; doi:10.3390/ijms24032960)
Supplement: Supplementary file 1 [file ijms-24-02960-s001.zip › ijms-2179751-supplementary.pdf]

SUPPLEMENTAL MATERIAL

Figure S1. Probenecid blunted VSMC migratory capacity induced by UA

A) Graph depicts the inhibitory effects of 10 and 20 μM P after 24 hour exposition to 9 mg/dl UA.
B) This effect was confirmed by AlexaFluor 594-Phalloidin staining. The yellow arrows point out F-actin cortical distribution in UA exposed cells, whereas it was absent in P pretreated cells. Magnification X400, scale bar= 50 μm. Results represent means ± SEM obtained from three/four independent experiments and are expressed as fold change to CTR.
***p < 0.0001 vs CTR; §§ p<0.001 vs UA treated cells.

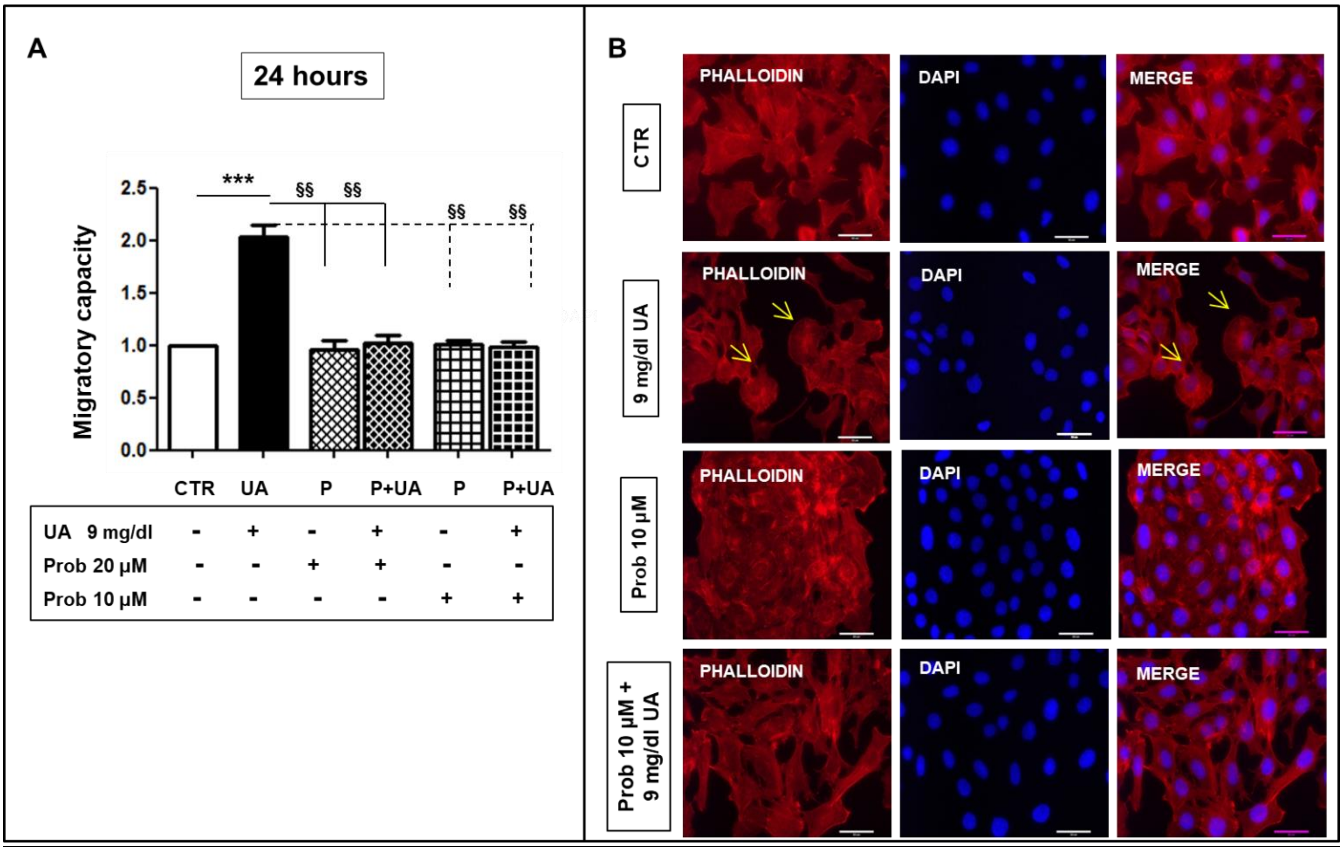

P=Probenecid; VSMC= Vascular smooth Muscle cells; UA=Uric Acid; ; CTR= control untreated cells.

Figure S2. Atrogin-1 and α-SMA expression after 48 hrs VSMC exposition to Probenecid and 9 mg/dl UA.

A) In UA treated cells, 10 µM P treatment down regulated Atro-1 mRNA, detected by rt-PCR.
B-C) Atro-1 protein expression, evaluated by immunofluorescence and reported as CTCF, was decreased. C) P induced changes in expression and distribution of Atro-1 (red) and α-SMA (green). Signal intensities were evaluated by Image J in at least 100 cells for condition from three independent experiments. rt-PCR results represent means ± SEM obtained from three/four independent experiments and are expressed as fold change to CTR.
* $p<0.05$ vs CTR, § $p<0.05$ vs UA treated cells. Magnification= X400, scale bar= 50 µm.

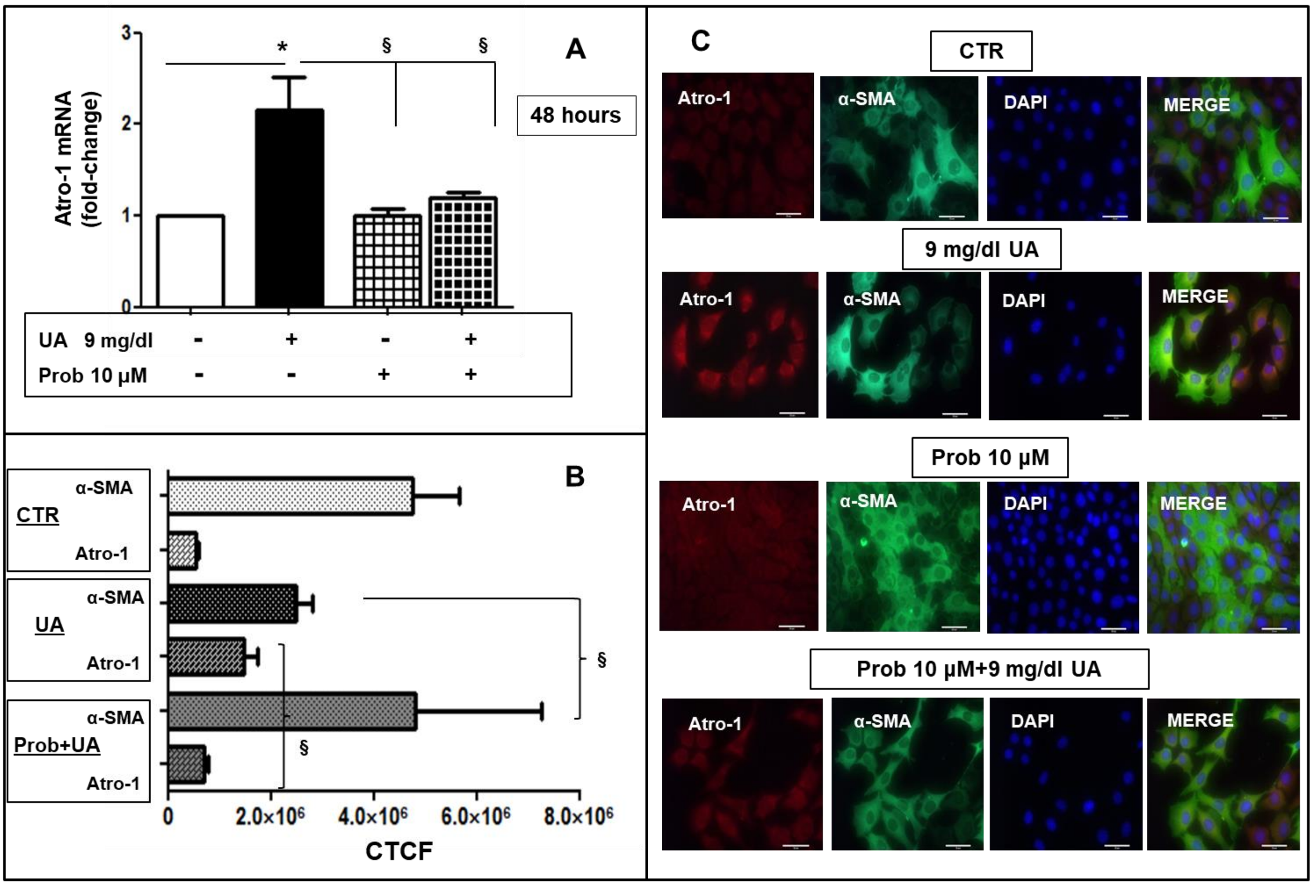

Atro-1=Atrogin-1; α-SMA= α-Smooth muscle actin; P=Probenecid; VSMC= Vascular Smooth Muscle Cells; UA=Uric Acid; rt-PCR= real time PCR; CTR= control untreated cells; CTCF= Corrected Total Cell Fluorescence; DAPI= 4′,6-diamidino-2-phenylindole.
